# Supplementary material for: Preparation and Characterization of Nifedipine-Loaded Dry Medium Internal-Phase Emulsions (Dry MIPEs) to Improve Cellular Permeability
Source: Pharmaceutics. 2022 Sep 1;14(9):1849. doi: 10.3390/pharmaceutics14091849 (PMC9502710; doi:10.3390/pharmaceutics14091849)
Supplement: Supplementary file 1 [file pharmaceutics-14-01849-s001.zip › pharmaceutics-1874057-supplementary.pdf]

pharmaceutics

MDPI

Supplementary Material: Preparation and Characterization of Nifedipine-loaded Dry Medium Internal-phase Emulsions (Dry MIPEs) to Improve Cellular Permeability

Sukannika Tubtimsri and Yotsanan Weerapol

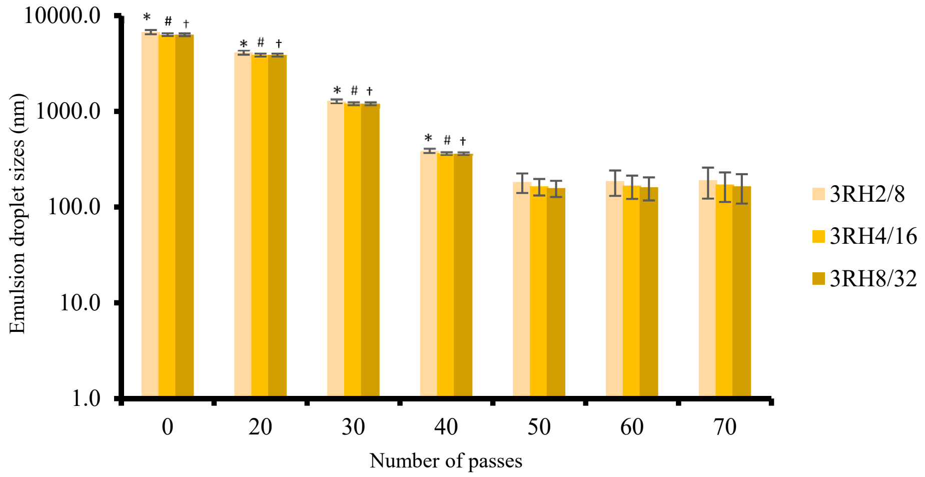

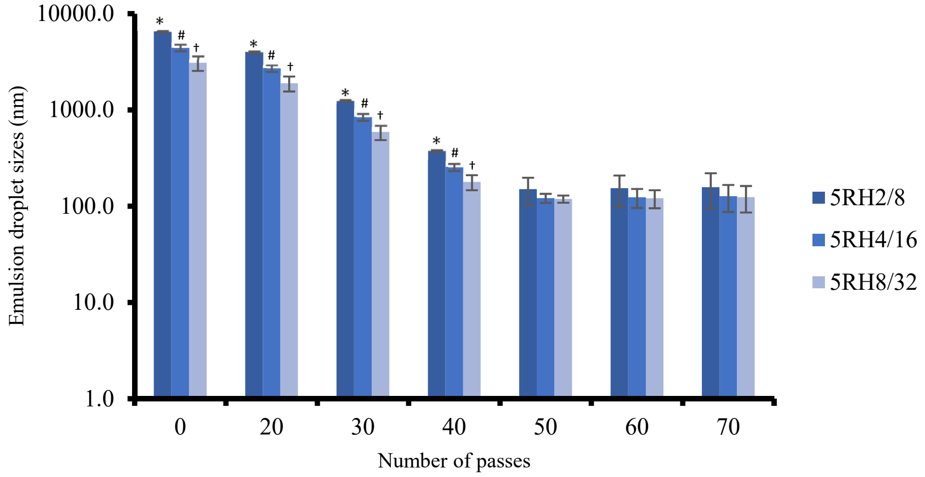

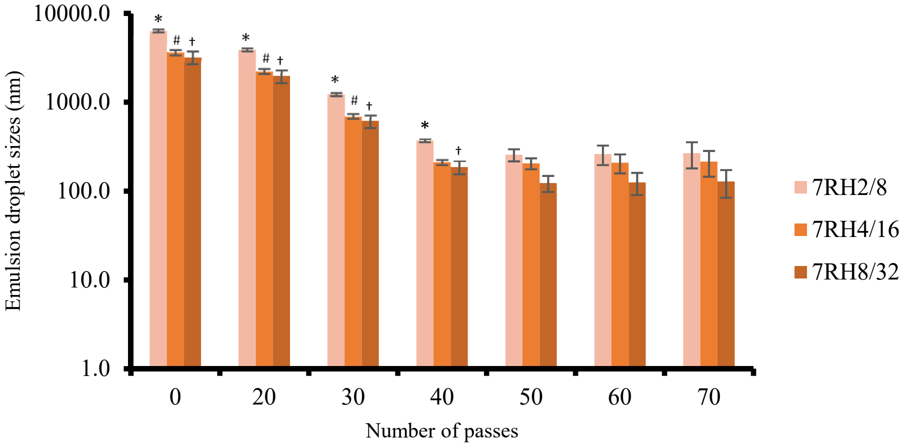

Figure S1. Emulsion droplet sizes after passing though the microfluidizer 0, 20, 40, 50, 60 and 70 passes. Values are means ± SD, n = 3. $^{*,\#,\dagger}$ $p < 0.05$ when compared to numbers of passes 50.

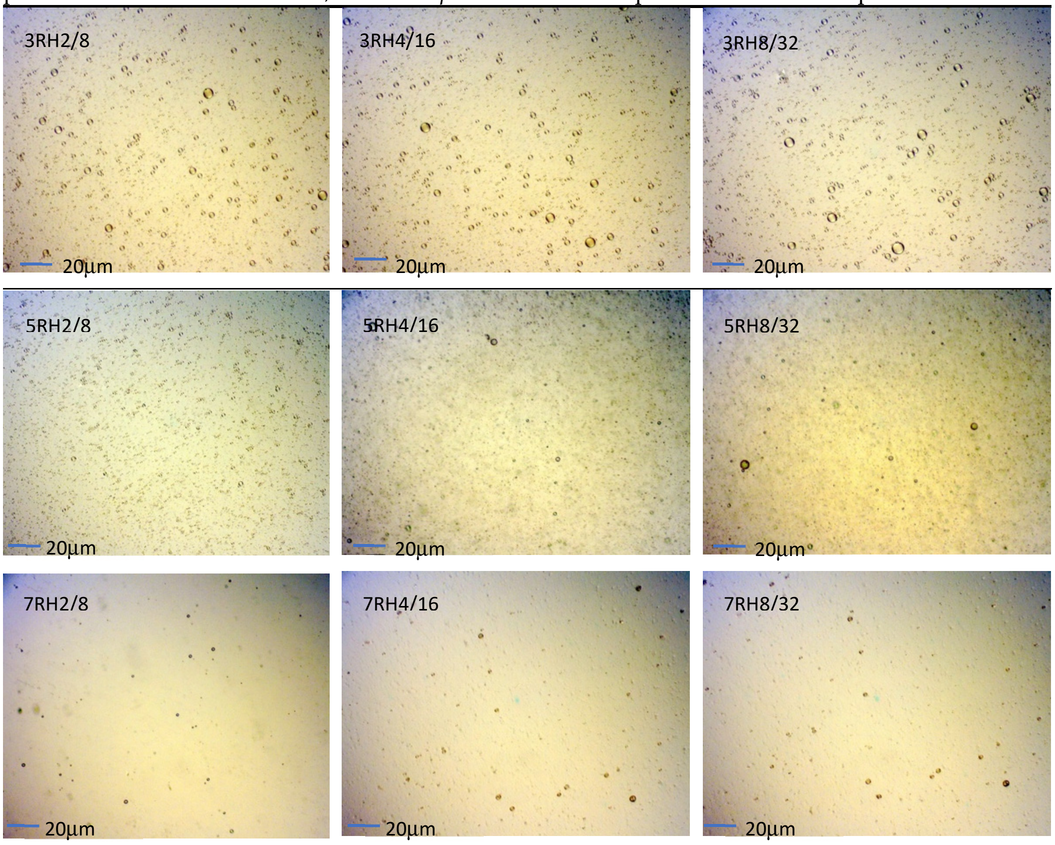

Figure S2. The images from optical microscopy (magnification, ×400) of diluted dry MIPEs with simulated gastric fluid (SGF).

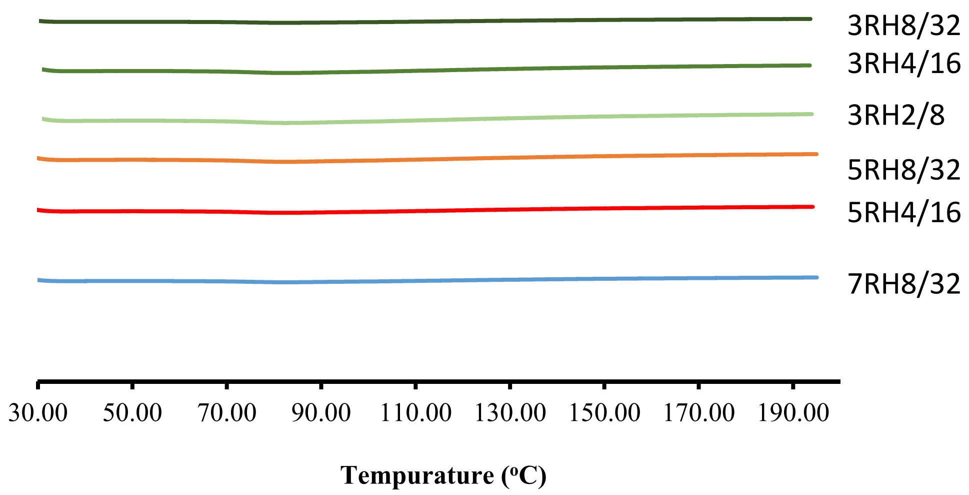

Figure S3. Differential scanning calorimetry (DSC) thermograms of 5RH4/16, 5RH8/32, and 7RH8/32 after storage under accelerated conditions (relative humidity 40° C/75%).

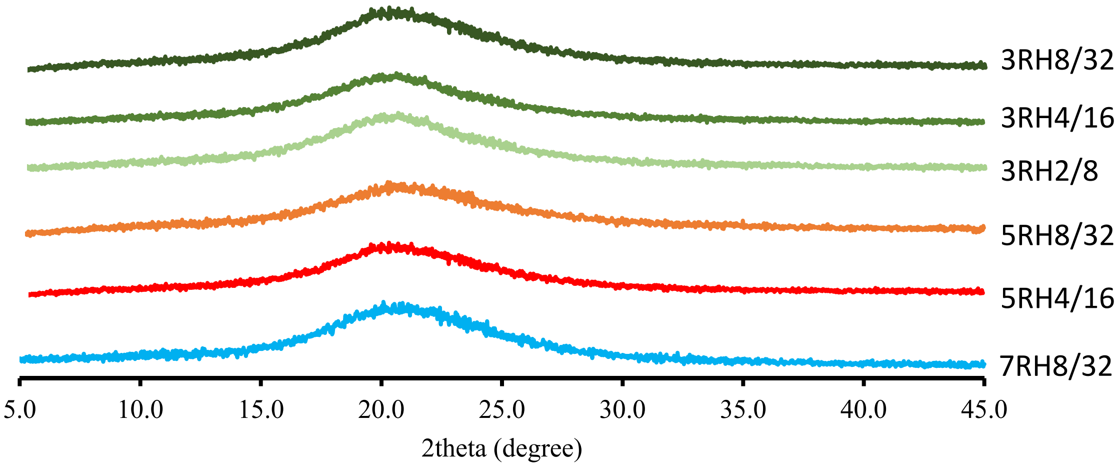

Figure S4. Powder X-ray diffraction patterns of 5RH4/16, 5RH8/32, and 7RH8/32 after storage under accelerated conditions (relative humidity 40° C/75%).

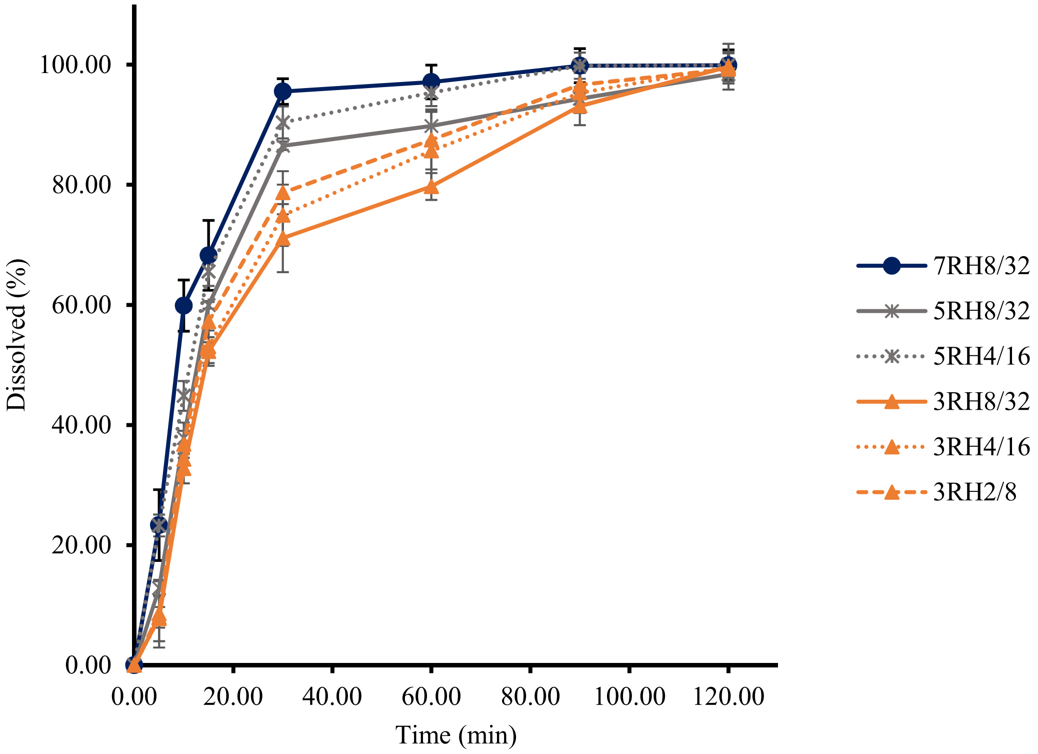

Figure S5. Dissolution of 5RH4/16, 5RH8/32, and 7RH8/32 after storage under accelerated conditions (relative humidity 40°C/75%).
